# Evaluation of a commercial synthetic computed tomography generation solution for magnetic resonance imaging‐only radiotherapy

**DOI:** 10.1002/acm2.13236

**Published:** 2021-05-27

**Authors:** A. Gonzalez‐Moya, S. Dufreneix, N. Ouyessad, C. Guillerminet, D. Autret

**Affiliations:** ^1^ Institut de Cancérologie de l’Ouest Angers France

**Keywords:** Hounsfield Unit, MR‐only treatment planning, synthetic computed tomography

## Abstract

**Purpose:**

To evaluate the Siemens solution generating Synthetic computed tomography (sCT) for magnetic resonance imaging (MRI)‐only radiotherapy (RT).

**Method:**

A retrospective study was conducted on 47 patients treated with external beam RT for brain or prostate cancer who underwent both MRI and CT for treatment planning. sCT images were generated from MRI using automatic bulk densities segmentation. The geometric accuracy of the sCT was assessed by comparing the Hounsfield Units (HU) difference between sCT and CT for bone structures, soft‐tissue, and full body contour. VMAT plans were computed on the CT for treatment preparation and then copied and recalculated with the same monitor units on the sCT using the AcurosXB algorithm. A 1%‐1mm gamma analysis was performed and DVH metrics for the Planning Target Volume (PTV) like the D_mean_ and the D_98%_ were compared. In addition, we evaluate the usability of sCT for daily position verification with cone beam computed tomography (CBCT) for 14 prostate patients by comparing sCT/CBCT registration results to CT/CBCT.

**Results:**

Mean HU differences were small except for the skull (207 HU) and right femoral head of four patients where significant aberrations were found. The mean gamma pass rate was 73.2% for the brain and 84.7% for the prostate and D_mean_ were smaller than 0.5%. Large differences for the D_98%_ of the prostate group could be correlated to low Dice index of the PTV. The mean difference of translations and rotations were inferior to 3.5 mm and 0.2° in all directions with a major difference in the anterior‐posterior direction.

**Conclusion:**

The performances of the software were shown to be similar to other sCT generation algorithms in terms of HU difference, dose comparison and daily image localization.

## 1 INTRODUCTION

The main reason for including magnetic resonance imaging (MRI) in the Radiation Therapy (RT) workflow is the higher soft tissue contrast compared to CT. MRI cannot be used directly for dose calculation because MR intensities correlate with proton densities and relaxation properties whereas dose calculation in treatment planning systems requires data on electron density from CT.^1^ Therefore, a combined workflow is used including both MRI for tissue segmentation and CT for dose calculation after MRI‐CT registration, which can introduce uncertainties estimated to be up to 2–5 mm.^2^ Interest is growing to use MRI as the only modality in RT to take advantage of its soft tissue contrast and remove inter‐modality registration uncertainties.^3^ Recent studies have investigated the feasibility of implementing an MR‐only workflow by synthetically generating CT images, called synthetic‐CT (sCT), directly obtained from MRI to enable dose calculation and position verification.^4^

Recently, SIEMENS HEALTHINEERS® commercialized a solution which generates sCT using a bulk density method. In this method, MRI voxel intensity information is translated to CT numbers and sCT images.^5^ Bulk density techniques either used a single homogeneous or a multiple tissue override, which improve the dose calculation but required long manual contouring.^6^ The imaging software provided by SIEMENS HEALTHINEERS® generates the sCT image using an automatic tissue classification method with five classes. To the best of our knowledge, there is no study evaluating the performances of this solution. The objective of the study was to evaluate the geometric accuracy, dose calculation and positioning performance of this commercial sCT approach in external beam radiotherapy for brain and prostate cancer.

## 2 MATERIALS AND METHODS

### 2.1 Patient data, image acquisition, and sCT generation

Retrospective analysis was performed using MRI and CT data from 47 patients (16 primary brain tumors and 31 prostate cancer). Approval for the study protocol was obtained from the medical research ethics committee and informed consent was obtained from all patients. See Table 1 for more information about RT volumes and prescribed doses.

**Table 1:** Volumes, prescribed doses and type of RT for prostate and brain cancer groups.

Brain (n = 16)	Target	Meningioma	2
		Glioma	13
		Craniopharyngioma	1
	Prescribed dose (Gy)‐ (Gy/fraction x fractions)	60 – (2 x 30)	9
		54 – (1,8 x 30)	2
		52,2 – (1,8 x 29)	1
		40 – (2,66 x 15)	4
	Brain RT	Alone	6
		Post‐operative	10
Prostate (n = 31)	Target	Prostate alone	6
		Prostate and vesicles	3
		Prostate, vesicles and lymph nodes	19
		Prostate and bone metastases	1
		Prostate cavity and pelvis	2
	Prescribed dose (Gy)‐ (Gy/fraction x fractions)	78 – (2 x 39)	26
		74 – (2 x 37)	1
		66 – (2 x 33)	2
		60 – (3 x 20)	2
	Prostate RT	Alone	29
		Post‐operative	2

Patients first underwent a CT acquisition with a SIEMENS SOMATOM Confidence RT scan. Then, MR images were acquired using a 1,5 T SIEMENS MAGNETOM AERA XJ MRI scan® with a mean time of 4 days (range 0–11) between the two imaging sessions. MRI sequences were acquired to generate sCT images in addition to routine sequences used for delineation (see Supplemental Table S1 and S2 for magnetic parameters details). The CT and MR images were acquired in the treatment position before RT. For brain imaging, the head was immobilized in a thermoplastic three‐point mask during both planning CT and MRI. Masks were marked at the CT session and patient repositioning at the MRI session was checked with a laser system. For prostate imaging, patients were positioned in a supine position on the provided flat table, with identical immobilization thanks to a knee support cushion. Patients were tattooed at the CT session and repositioning at the MRI session was checked with a laser system.

**Fig. 1:**
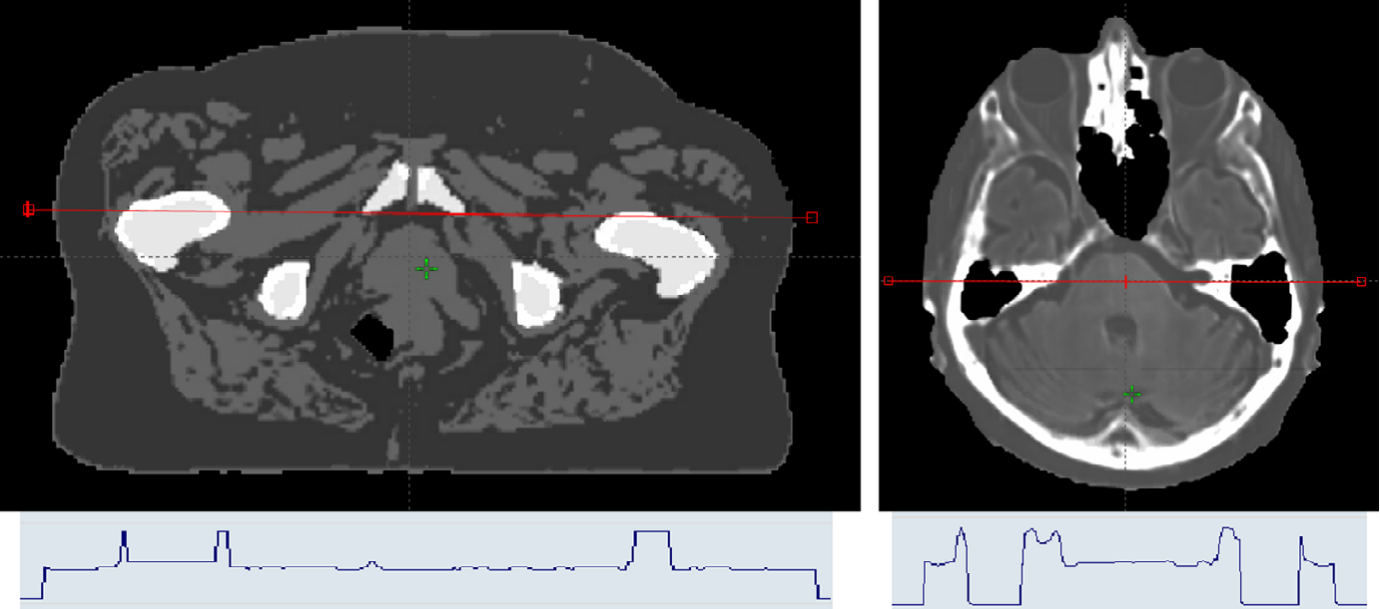
Example of s‐CT images obtained for the pelvis (Left) and the brain (Right) with a HU profile (red line on the s‐CT) stressing the segmented (left) and continuous (right) HU affectation.

After acquisition, the sCT was generated using an automatic tissue classification method. For the brain, each voxel was assigned with a probability of belonging to each of five tissue classes (fat, water, white matter, grey matter, and bone). A continuous spectrum from −1000 to 1096 HU led to sCT image generation. For the pelvis, using the Dixon MR images reconstruction, the content of the MR image was categorized into five classes. Then, each voxel was assigned to corresponding HUs to generated sCT with discrete HU distribution: air (−1000 HU), fat (−75 HU), water (0 HU), spongy bone (204 HU), and cortical bone (1170 HU). The soft tissue of bladder, rectum, and prostate is assigned 0 HU like water (Fig. 1).

### 2.2 Structures delineation

**Fig. 2:**
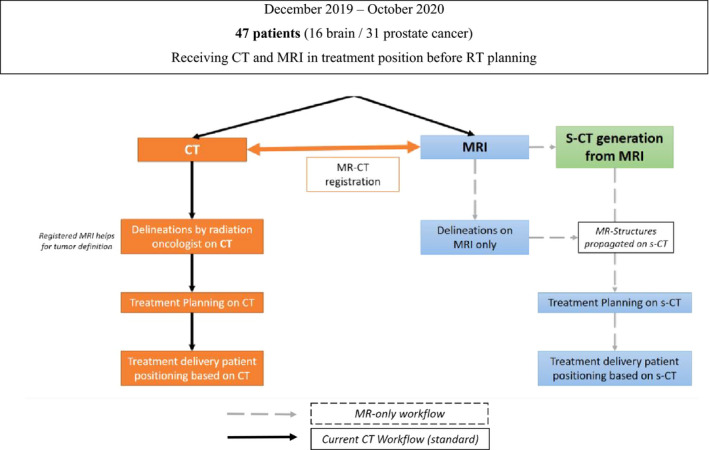
Study workflow evaluating the feasibility of MRI only RT.

The study and standard workflows are described in Fig. 2. Each MRI was rigidly registered to the corresponding CT. For brain treatment, PTVs in CT RT structures and MR RT structures were identical because routinely, PTV is delineated on the MR images and then reported on the planning CT. For prostate treatment, MRI was rigidly registered to CT focusing on a match of the prostate. PTVs can differ between the MR RT structures and the CT RT structures as the standard practice is still based on CT for prostate delineations. Contours on MR images were performed retrospectively by another observer and reviewed by an experienced radiation oncologist. Since sCT images share the same frame with the underlying MRI, MR delineations could be propagated to the sCT without introducing systematic error.

### 2.3 Geometric accuracy

A comparison was made between the HUs of the sCT and the HUs of the reference CT scan for bone structures, soft‐tissue and body contour in prostate and brain groups. Bone segments were generated by thresholding the respective images at 100 HU, followed by a morphological hole filling. Soft tissue segments were generated by thresholding the sCTs and CTs at −100 HU and subtracting the previously generated bone structures. Body contour segments were similarly generated by thresholding at −350 HU, followed by a morphological hole‐filling. Mean HU were extracted in each volume and differences were calculated between the CT and sCT for each patient.

### 2.4 Dose comparison

For each PTV, a 2‐arcs (or 2‐partial‐arcs) VMAT plan was optimized and calculated with the AcurosXB algorithm with a 2‐mm grid size based on the CT associated with the CT RT structures, as in clinical practice. Prostate localization could be associated up to 3 PTVs (prostate, prostate and vesicles, pelvis), each associated to a different plan. Plans were then copied on the sCT associated with the MR RT structures. Calculation on the sCT used the same MU values, and no re‐optimization was performed. The same CT calibration curve was used for converting HU into electron densities for the CT and sCT. Using the Radiological Imaging Technology software (Radimage®), the mean dose difference and the 1%‐1mm gamma index were computed with a cut‐off isodose of 2% in the axial, frontal, and sagittal slices crossing the isocenter. A DVH analysis was finally conducted on the PTV structures by reporting the D_2%_, D_95%_, D_98%_, and D_mean_. Mean differences, gamma, and DVH analysis were conducted relative to the prescribed dose.

### 2.5 Positioning performance

The accuracy of using sCT for position verification was checked for 14 prostate patients by comparing sCT/ CBCT registration results to CT/CBCT registrations, during 3 fractions (the first, mid, and last treatment day). Fusions were estimated within a box including bony pelvic anatomy, excluding femoral heads. The sCT and CT images were registered using auto‐match towards CBCT images with a manual correction as in routine. Positioning accuracy was calculated to six degrees of freedom (3 translational and 3 rotational axes) in left‐right (LR), anterior‐posterior (AP) and superior‐inferior (SI) directions. Each vector was calculated using either the CT or the sCT as reference and mean differences were calculated using the following equation:

$$\Delta V=\Delta V_{\text{CBCT}/\text{CT}}‐\left(\Delta V_{\text{CBCT}/\text{sCT}}+\Delta V_{\text{CT}/\text{sCT}}\right).$$

where $\Delta V_{\text{CT}/\text{sCT}}$ is the intrinsic offset between CT and sCT.

## 3 RESULTS

Three prostate patients were excluded from the study (2 having an artefact or a prothesis on MRI and 1 because MRI did not encompass the entire body).

### 3.1 Geometric accuracy

Average HU value and mean differences between CT and sCT are given in Table 2.

**Table 2:** Mean absolute HU and mean difference between s‐CT and CT for different brain and pelvic tissue segments.

Location	Segments	CT HU value Mean (SD)	s‐CT HU value Mean (SD)	ΔHU (CTUH – s‐CTUH) Mean (SD)
Brain	Entire head	105.8 ± 18.0	92.3 ± 13.2	13.5 ± 4.8
	Brain	43.6 ± −2.8	49.7 ± 6.8	‐6.1 ± −9.6
	Skull	751.5 ± 83.8	544.8 ± 51.3	206.7 ± 32.5
Prostate	Entire pelvis	‐1.2 ± 14.0	‐22.4 ± 7.8	21.2 ± 6.2
	Soft tissues	‐2.1 ± 18.9	‐40.2 ± 7.3	38.1 ± 11.7
	Femoral bones	332.5 ± 41.0	306.4 ± 33.3	26.1 ± 7.7

Across 16 brain cancer patients, the largest differences were found at the body contours, bone/soft tissues interfaces and the bones, similar to those reported with a large discrepancy in the regions with high CT number.^7^ Skull has very dense bones on CT (up to 2000 HU) and HUs of bones were on average lower on the sCT as the method is based on a continuous spectrum up to 1096 HU only.

Across 31 prostate cancer patients, mean HU differences for the entire pelvis were greater to those reported ranging from 1.9 ± 6.6 to 7.7 ± 7.3.^8^, ^9^ HU differences for bones were also greater to those reported ranging from 7.8 ± 46.0 HU to 18.2 ± 24.5 HU.^8^, ^10^ In our study, worst HU value agreement was found for 4 patients (mean HU difference 52.4 ± 19.8) having significant aberrations in the right femoral head (unrealistic femur reconstructions) on sCT images related to SIEMENS®’ algorithm error (see supplementary Figure S1). Except those patients, the mean HU difference for femoral heads is improved (19.8 ± 10.7 HU).

### 3.2 Dose comparison

**Fig. 3:**
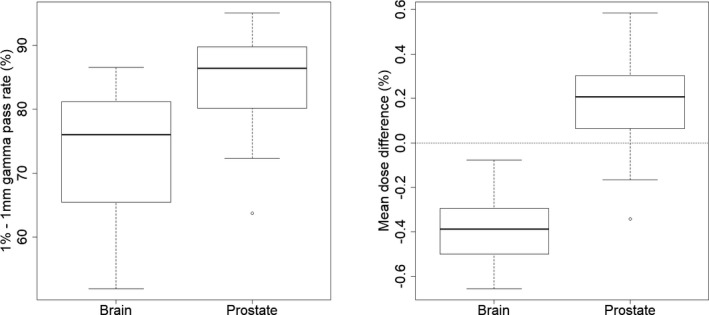
Mean gamma pass rate (left) and mean dose difference (right) over the axial, frontal and sagittal slices crossing the isocenter considering a 2 % cut‐off isodose.

**Fig. 4:**
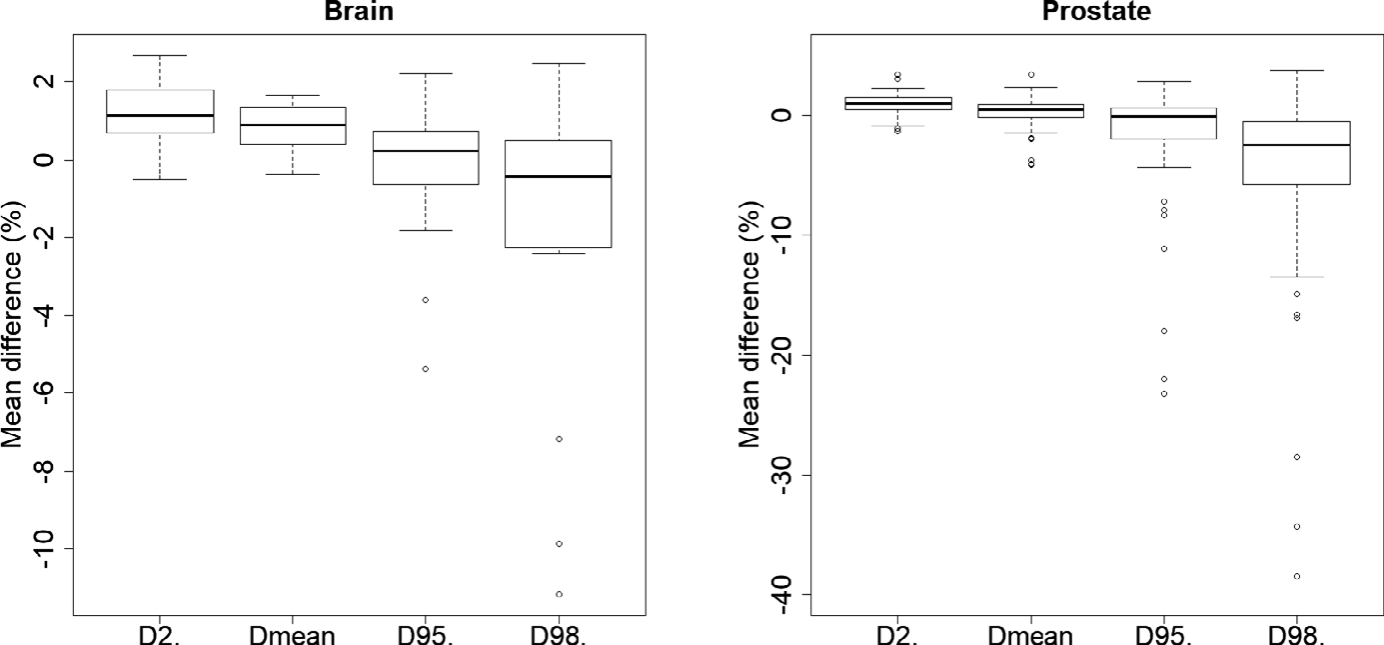
Results of the DVH analysis for the brain (left) and prostate (right) localization.

Results of the gamma pass rate and mean dose difference analysis are plotted in Fig. 3. For the brain, the mean 1%‐1mm gamma pass rate was 73.2% [51.8%–86.5%], lower than other reference^7^, ^11^ and mean dose difference showed an underestimation of the dose calculated on the sCT (−0.4 ± 0.2%). Main deviations were found near the edge of the outer contour, which can slightly differ between the CT and sCT although patients were immobilized with a mask. This could also be explained by the deviation of the HU observed in the previous section for the skull region. For the prostate, a higher mean gamma pass rate was observed with a mean of 84.7% [63.7% ‐ 95%] and the mean dose difference was close to zero (0.2 ± 0.2%). The dose differences obtained in this study are in line with previously published studies on prostate cancer patients, which reported dose differences within 1%.^8^, ^12^, ^13^, ^14^ Comparisons of the PTV metrics are plotted in Fig. 4. For the brain, differences for the D_95%_ (−0.1 ± 2.3%) and the D_98%_ (−1.6 ± 4.1%) were observed. The dose underestimations were found for PTVs of close proximity to air cavities where the sCT assigned −1000 HU resulting in deviations of more than 10% for D_98%_. Deviations were observed for the prostate metrics, also associated to larger standard deviations: D_2%_ = 0.9 ± 1.0%, D_mean_ = 0.3 ± 1.4%, D_95%_ = −2.6 ± 7.9% and D_98%_ = −5.8 ± 11.3%. This can be explained by the fact that plans were optimized on CT and simply recalculated on the sCT. Since PTV prostate can differ between the CT RT and the MR RT structures, the VMAT plan calculated on the sCT without re‐optimization can generate dose distributions which do not nicely encompass the PTV of the MR RT structure.

### 3.3 Positioning performance

**Fig. 5:**
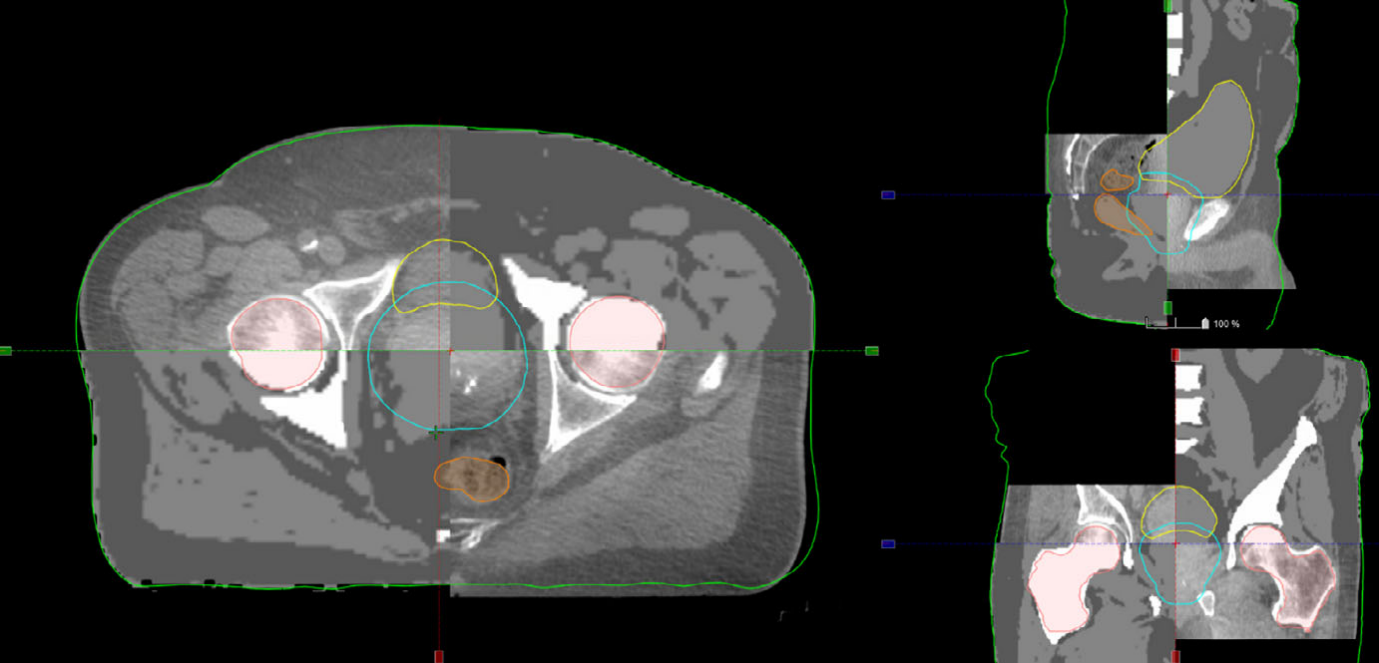
sCT / CBCT comparison. The following structures delineated on the MRI are displayed: outer contour, femoral heads, bladder, rectum and prostate PTV.

42 CBCTs were used for registration with 3 CBCT per patient over the 14 considered patients. An illustration of the sCT/CBCT comparison is displayed on Fig. 5. The mean differences in shift translations between CBCT‐to‐CT and CBCT‐to‐sCT registrations were 3.5 ± 7.7 mm in the AP, 0.2 ± 1.8 mm in the SI and −0.8 ± 2.9 mm in the LR directions. The mean differences in shift rotations were smaller than 0.2° in all directions (Table 3). Largest differences were observed in AP may be due to patient position or non‐compliance with rectum preparation at the MRI and CT simulations. Results were in agreement with others reporting millimeters shift translations and rotation. Tyagi et al. reported mean differences are within 1.0 ± 0.8 mm for the LR, 1.0 ± 0.9 mm for the AP and less than 0.5 ± 0.9 mm for the SI direction.^15^ Maspero et al. reported mean differences less than 0.5 mm and 0.5° in all the directions, except AP, where a systematic difference within 1.0 mm was found.^12^ Kemppainen et al. reported a 0.5 mm systematic difference in the AP direction.^16^

**Table 3:** Mean set‐up correction differences between s‐CT with CBCT and CT with CBCT with six degrees of freedom.

Direction	∆V Translations (mm)	∆V Rotations (°)
X (Left‐ Right)	‐0.8 ± 2.9	‐0.2 ± 0.4
Y (Anterior‐posterior)	3.5 ± 7.7	0.0 ± 0.3
Z (Superior‐inferior)	‐0.2 ± 1.8	‐0.1 ± 0.3

## 4 DISCUSSION

For the brain, mean HU differences were small, except for the bones. The high HU of the skull is known to be underestimated by sCT.^7^ For the prostate, mean HU differences observed for the entire pelvis were greater than those reported in the literature.^8^, ^9^ Significant aberrations exist on sCT images reconstruction, currently under investigation by SIEMENS®.

**Fig. 6:**
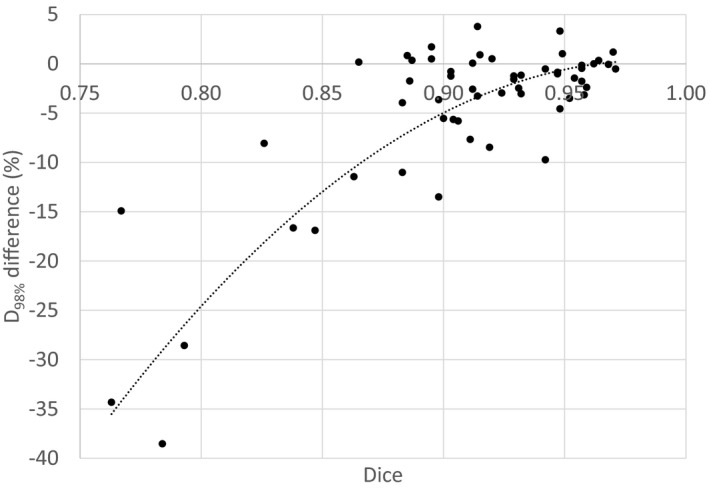
Difference on the D98% between the s‐CT and CT represented as a function of the DICE. Each point represents a plan associated to a PTV (either prostate, prostate and seminal vesicles or pelvis). Dashed line represents a two order polynomial.

The dosimetric comparison of plans optimized on the CT and calculated on the CT and sCT for the brain showed similar results in the PTV region, except when air cavities were in close proximity. For the prostate group, large differences were observed for the D_95%_ and D_98%_. However, the dose comparison conducted encompasses both differences linked to the change of modality (CT/sCT) and to the change of PTV contours (CT and MRI respectively). The DICE index of two volumes is defined as the intersection of the volumes divided by the union of the volumes.^17^ Figure 6 shows the D_98%_ metric observed for the prostate plans as a function of the DICE index of the PTV between CT RT and MR RT structures. Largest differences are observed for lower DICE indexes and can thus be attributed to patient repositioning between the CT and MRI. The Pearson correlation coefficient between D_98%_ and DICE index was 0.70 (*P*‐value = 1.2e‐9, 95% confidence interval: 0.54‐0.81) confirming the correlation of the two metrics. For DICE index higher than 0.9 (representing a good agreement between PTVs delineated on the CT and MRI), the mean D_98%_ difference is −2.3 ± 3.4% and the mean D_95%_ is −0.3 ± 2.0%. These differences can be attributed to the change of modality between calculation on CT and on the sCT. A similar trend was observed for the D_95%_. This high sensibility of the dosimetric analysis could also be linked to the algorithm used : a type “c” algorithm, with similar performances to those of Monte Carlo algorithm and very sensitive to heterogeneities.^18^

In this work, we found that sCT can be used to set‐up corrections with mean difference of translations and rotations over 14 prostate cancer patients (42 CBCT) inferior to 3.5 mm and 0.2° respectively, with a major difference in AP, which favorably compares with others.^12^, ^15^, ^16^

Different sCT generation methods have been proposed in the literature. These techniques recently underwent significant changes with the emergence of deep learning methods. Comparison between approaches is difficult because of differences in the size of dataset, tumor location, MRI sequences, image registration modalities and performance metrics used to evaluate the method. There is no literature consensus to implement MR as the only imaging modality for RT. Guidelines would be interesting to standardize practice and to ensure that MRI acquired beyond the control of the RT department is appropriate to use.

## 5 CONCLUSION

This is the first study evaluating the sCT generation method proposed by SIEMENS® for brain and prostate locations using a high sensibility algorithm for the dosimetric analysis (AcurosXB). The performances of the software were evaluated in terms of HU difference, dose comparison and daily image localization and showed reasonable deviations between CT and sCT. The largest differences of the dose comparison could be related to patient repositioning between the CT and MRI.

## Author Contribution

D. Autret, S. Dufreneix and C. Guillerminet were responsible for the study design. A. Gonzalez‐Moya and N. Ouyessad were responsible for the data acquisition, analysis and interpretation. A. Gonzalez‐Moya and S. Dufreneix drafted the article. All authors read and approved the final manuscript.

## Conflict of interest

None.

## Supporting information


**Fig S1** s‐CT image for the pelvis with unrealistic femur reconstruction during s‐CT generation

**Table S1** MRI simulation scanning parameters for prostate group including current sequences used for anatomic segmentation (black) and additional sequences needed for s‐CT generation (blue)

**Table S2** MRI simulation scanning parameters for brain group including current sequences used for anatomic segmentation (black) and additional sequences needed for s‐CT generation (blue)

## References

[1] Wafa B , Moussaoui A . A review on methods to estimate a CT from MRI data in the context of MRI‐alone RT. Med Technol J. 2018;2:150–178.

[2] Nyholm T , Nyberg M , Karlsson MG , Karlsson M . Systematisation of spatial uncertainties for comparison between a MR and a CT‐based radiotherapy workflow for prostate treatments. Radiat Oncol. 2009;4:54. 19919713 10.1186/1748-717X-4-54 PMC2781017

[3] Owrangi AM , Greer PB , Glide‐Hurst CK . MRI‐only treatment planning: Benefits and challenges. Phys Med Biol. 2018;63:5TR01. 10.1088/1361-6560/aaaca4 PMC5886006 29393071

[4] Johnstone E , Wyatt JJ , Henry AM , et al, Systematic review of synthetic computed tomography generation methodologies for use in magnetic resonance imaging‐only radiation therapy. Int J Radiat Oncol. 2018;100:199–217. 10.1016/j.ijrobp.2017.08.043 29254773

[5] Eilertsen K , Vestad LNTA , Geier O , Skretting A . A simulation of MRI based dose calculations on the basis of radiotherapy planning CT images. Acta Oncol. 2008;47:1294–1302. 18663645 10.1080/02841860802256426

[6] McCallum HM , Andersson S , Wyatt JJ , Richmond N , Walker CP , Svensson S . Technical Note: Efficient and accurate MRI‐only based treatment planning of the prostate using bulk density assignment through atlas‐based segmentation. Med Phys. 2020;47:4758–4762. 32682337 10.1002/mp.14406

[7] Paradis E , Cao Y , Lawrence TS , et al, Assessing the dosimetric accuracy of magnetic resonance‐generated synthetic CT images for focal brain VMAT radiation therapy. Int J Radiat Oncol. 2015;93:1154–1161. 10.1016/j.ijrobp.2015.08.049 PMC4654706 26581151

[8] Siversson C , Nordström F , Nilsson T , et al, Technical Note: MRI only prostate radiotherapy planning using the statistical decomposition algorithm: MRI‐only radiotherapy planning using the statistical decomposition algorithm. Med Phys. 2015;42:6090–6097. 26429284 10.1118/1.4931417

[9] Cronholm C . MRI only radiotherapy planning using the transfer function estimation algorithm. (2020). MRI Planner White Paper ‐ Spectronic Medical.

[10] Dowling JA , Lambert J , Parker J , et al, An atlas‐based electron density mapping method for magnetic resonance imaging (MRI)‐alone treatment planning and adaptive MRI‐based prostate radiation therapy. Int J Radiat Oncol. 2012;83:e5–e11. 10.1016/j.ijrobp.2011.11.056 22330995

[11] Dinkla AM , Wolterink JM , Maspero M , et al, MR‐only brain radiation therapy: Dosimetric evaluation of synthetic CTs generated by a dilated convolutional neural network. Int J Radiat Oncol. 2018;102:801–812. 10.1016/j.ijrobp.2018.05.058 30108005

[12] Maspero M , Tyyger MD , Tijssen RHN , et al, Feasibility of magnetic resonance imaging‐only rectum radiotherapy with a commercial synthetic computed tomography generation solution. Phys Imaging Radiat Oncol. 2018;7:58–64. 33458406 10.1016/j.phro.2018.09.002 PMC7807733

[13] Persson E , Gustafsson C , Nordström F , et al, MR‐OPERA: A multicenter/multivendor validation of magnetic resonance imaging‐only prostate treatment planning using synthetic computed tomography images. Int J Radiat Oncol. 2017;99:692–700. 10.1016/j.ijrobp.2017.06.006 28843375

[14] Christiansen RL , Jensen HR , Brink C . Magnetic resonance only workflow and validation of dose calculations for radiotherapy of prostate cancer. Acta Oncol. 2017;56:787–791. 28464739 10.1080/0284186X.2017.1290275

[15] Tyagi N , Fontenla S , Zhang J , et al, Dosimetric and workflow evaluation of first commercial synthetic CT software for clinical use in pelvis. Phys Med Biol. 2017;62:2961–2975. 27983520 10.1088/1361-6560/aa5452 PMC5541676

[16] Kemppainen R , Suilamo S , Ranta I , et al, Assessment of dosimetric and positioning accuracy of a magnetic resonance imaging‐only solution for external beam radiotherapy of pelvic anatomy. Phys Imaging Radiat Oncol. 2019;11:1–8. 33458269 10.1016/j.phro.2019.06.001 PMC7807675

[17] Dice LR . Measures of the amount of ecologic association between species. Ecology. 1945;26:297–302.

[18] Ojala JJ , Kapanen MK , Hyödynmaa SJ , Wigren TK , Pitkänen MA . Performance of dose calculation algorithms from three generations in lung SBRT: Comparison with full Monte Carlo‐based dose distributions. J Appl Clin Med Phys. 2014;15:4–18. 10.1120/jacmp.v15i2.4662 PMC5875463 24710454

