# Supplementary material for: Evaluation of a commercial synthetic computed tomography generation solution for magnetic resonance imaging‐only radiotherapy
Source: J Appl Clin Med Phys. 2021 May 27;22(6):191–7. doi: 10.1002/acm2.13236 (PMC8200507; doi:10.1002/acm2.13236)

Supplemental Figure 1: s-CT image for the pelvis with unrealistic femur reconstruction during s-CT generation


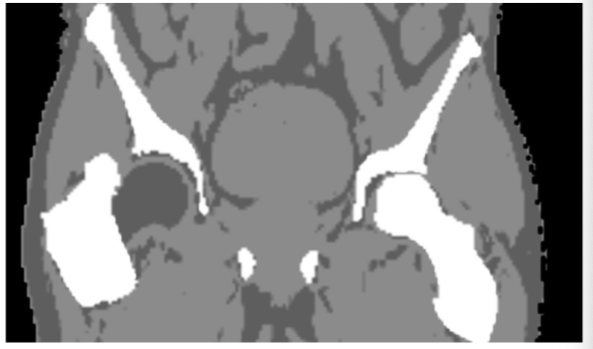

Supplement: Supplementary file 1 — Fig S1 s‐CT image for the pelvis with unrealistic femur reconstruction during s‐CT generation [file ACM2-22-191-s002.docx]
