# Supplementary material for: Evaluation of a commercial synthetic computed tomography generation solution for magnetic resonance imaging‐only radiotherapy
Source: J Appl Clin Med Phys. 2021 May 27;22(6):191–7. doi: 10.1002/acm2.13236 (PMC8200507; doi:10.1002/acm2.13236)
Supplement: Supplementary file 2 — Table S1 MRI simulation scanning parameters for prostate group including current sequences used for anatomic segmentation (black) and additional sequences needed for s‐CT generation (blue) [file ACM2-22-191-s001.pdf]

Supplemental Table 1: MRI simulation scanning parameters for prostate group including current sequences used for anatomic segmentation (black) and additional sequences needed for s-CT generation (blue)

Sequence	Acquisition Time (min)	BW (Hz/Px)	TE (ms)	TR (ms)	FA (°)	Resolution (mm x mm x mm)	ST (mm)	FOV (mm)
**AX T2 SPACE 3D**	6.45	558	132	1600	170	1.0 x 1.0 x 1.0	1	450
**T2 AX BLADE 2D**	5.17	446	78	6450	150	0.8 x 0.8 x 3.0	3	240
**DIXON 3D**	2.15	1010	2.39	6.27	15	2.0 x 2.0 x 2.0	2	448
