# Supplementary material for: Evaluation of a commercial synthetic computed tomography generation solution for magnetic resonance imaging‐only radiotherapy
Source: J Appl Clin Med Phys. 2021 May 27;22(6):191–7. doi: 10.1002/acm2.13236 (PMC8200507; doi:10.1002/acm2.13236)
Supplement: Supplementary file 3 — Table S2 MRI simulation scanning parameters for brain group including current sequences used for anatomic segmentation (black) and additional sequences needed for s‐CT generation (blue) [file ACM2-22-191-s003.pdf]

Supplemental Table 2: MRI simulation scanning parameters for brain group including current sequences used for anatomic segmentation (black) and additional sequences needed for s-CT generation (blue)

Sequence	Acquisition Time	BW (Hz/Px)	TE (ms)	TR (ms)	FA (°)	Resolution (mm x mm x mm)	ST (mm)	FOV (mm)
**SAG T1-3D MPRAGE with GADOLINIUM**	4.59	150	2.87	2200	8	1.0 × 1.0 × 1.0	1	255
**SAG FLAIR 3D SPACE**	5.18	592	366	6000		1.0 × 1.0 × 1.0	1	256
***T2 SPACE* SAG 3D**	5.32	723	99	1900	160	1.0 × 1.0 × 1.0	1	256
**PETRA 3D** **(***Bones)*	2.57	401	0.07	3.33	6	1.5 × 1.5 × 1.5	1.5	480
**T1 VIBE DIXON 3D** *(Water &fat)*	2.07	500	2.39	6.83	10	1.5 × 1.5 × 1.5	1.5	288
**FLASH Gradient Echo 2D***(Vessels)*	2.55	320	3.50	7	20	1.5 × 1.5 × 3.0	3	288
